# Therapeutic efficacy of combined BRAF and MEK inhibition in metastatic melanoma: a comprehensive network meta-analysis of randomized controlled trials

**DOI:** 10.18632/oncotarget.4375

**Published:** 2015-06-08

**Authors:** Ruiqin Mai, Songxia Zhou, Weixiang Zhong, Siming Rong, Zhichao Cong, Yunxian Li, Qizhi Xie, Huanming Chen, Xiaoyun Li, Shuhui Liu, Yabin Cheng, Yuanshen Huang, Youwen Zhou, Guohong Zhang

**Affiliations:** ^1^ Department of Laboratory Medicine, The First Affiliated Hospital of Shantou University Medical College, Shantou, Guangdong, China; ^2^ Department of Pathology, Shantou University Medical College, Shantou, Guangdong, China; ^3^ Department of Pathology, The First Affiliated Hospital, College of Medicine, Zhejiang University, Hangzhou, Zhejiang, China; ^4^ Department of Dermatology and Skin Science, University of British Columbia, Vancouver, British Columbia, Canada

**Keywords:** combing BRAF and MEK inhibition, targeted therapy, melanoma

## Abstract

**Background:**

Several recent randomized clinical trials have preliminarily demonstrated that initial targeted therapy with combined BRAF and MEK inhibition is more effective in metastatic melanoma (MM) than single agent. To guide therapeutic decisions, we did a comprehensive network meta-analysis to identify evidence to robustly support whether combined BRAF and MEK inhibition is the best initial targeted therapeutic strategy for patients with MM.

**Methods:**

The databases of PubMed and trial registries were researched for randomized clinical trials of targeted therapy. Data of outcome were extracted on progression-free survival (PFS), objective response rate (ORR), and overall survival (OS). Network meta-analysis using a Bayesian statistical model was performed to evaluate relative hazard ratio (HR) for PFS and OS, odds ratio (OR) for ORR.

**Results:**

Finally, 16 eligible trials comprising 5976 participants were included in this meta-analysis. PFS were significantly prolonged in patients who received combined BRAF-MEK inhibition compared with those who received BRAF inhibition (HR: 0.58, 95%CI: 0.51-0.67, *P* < 0.0001) or MEK inhibition alone (HR: 0.29, 95%CI: 0.22-0.37, *P* < 0.0001). Combined BRAF-MEK inhibition also improved the OS over BRAF inhibition (HR: 0.67, 95%CI: 0.56-0.81, *P* < 0.0001) or MEK inhibition alone (HR: 0.48, 95%CI: 0.36-0.65, *P* < 0.0001). The ORR was superior in combined BRAF and MEK inhibition comparing with BRAF inhibition (OR: 2.00, 95%CI: 1.66-2.44, *P* < 0.0001) or MEK inhibition alone (OR: 20.66, 95%CI: 12.22-35.47, *P* < 0.0001).

**Conclusions:**

This study indicates that concurrent inhibition of BRAF and MEK improved the most effective therapeutic modality as compared as single BRAF or MEK inhibition for patients with MM.

## INTRODUCTION

Metastatic melanoma (MM) used to be a fatal disease with an average survival of 7 months after diagnosis, since treatment options were limited. The discovery of driver oncogenic mutations of BRAF (eg.V600E, V600K) opens a new era in targeted therapy for MM. Indeed, the potent and specific BRAF (eg. dabrafenib, vemurafenib and sorafenib) inhibition, as compared with chemotherapy, have provided considerable clinical benefits including progression-free survival (PFS), overall survival (OS) and objective response rate (ORR) in patients with MM [1, 2]. However, most patients relapsed about 7 months after targeted therapy and approximately 14-26% of patients have development of secondary cutaneous squamous cell carcinoma and hyperkeratotic lesions within the first 2-3 months after BRAF inhibition [3]. Mechanism of acquired resistance commonly involves reactive MAPK pathway through mutant BRAF amplification and mutations activating RAS and MEK [4]. Therefore, downstream MAPK inhibition, such as MEK inhibition, was recognized as a promising target option. In fact, MEK inhibition (eg. trametinib) improved overall survival of MM patients with BRAF V600 mutation and not previously treated with BRAF inhibitors compared with chemotherapy [5]. In addition, the cutaneous adverse effects, such as cutaneous squamous cell carcinoma resulted by BRAF-inhibitor–induced paradoxical activation of the MAPK pathway in keratinocytes with upstream activation of signaling by preexisting RAS mutations [6, 7], which can be blocked with the addition of a MEK inhibition. Thus, combinative targeting the MAPK pathway via BRAF and MEK seem to provide greater clinical efficacy and reduce the adverse effects compared with BRAF inhibition alone.

Concurrent targeting BRAF and MEK has been considered the possibility to enhance tumor growth inhibition, delay acquired resistance, and abrogate paradoxical activation of the MAPK pathway in preclinical models of melanoma [6]. Recently several randomized controlled trials are on the way to evaluate efficacy of combined BRAF and MEK inhibition, such as the BRAF inhibitor dabrafenib and the MEK inhibition trametinib, have demonstrated superior response rate and prolonged survival [8-11]. However, the optimum treatment remains controversial and the feedback is not encouraged in term of the modestly enhanced, therapeutic efficacy [12]. In other way, it is difficult to integrate information on the relative efficacy compared with other combination treatments such as BRAF-chemotherapy, MEK-chemotherapy, and MEK alone. To establish the optimum treatment for MM, we did a random-effects network meta-analysis to compare combined BRAF and MEK inhibition in term of PFS, OS and ORR, respectively.

## RESULTS

### Eligible trials

**Figure 1:**
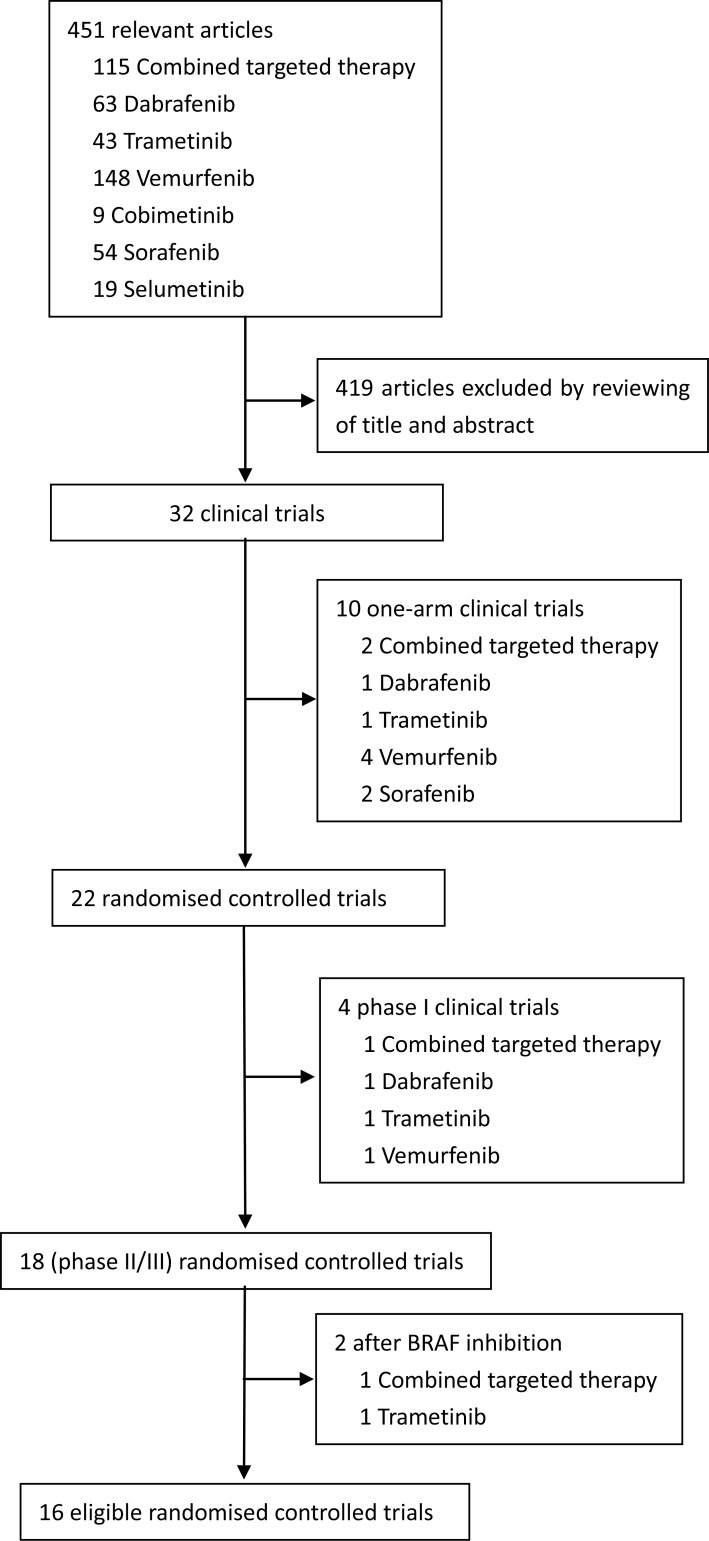
Study flow chart

**Figure 2:**
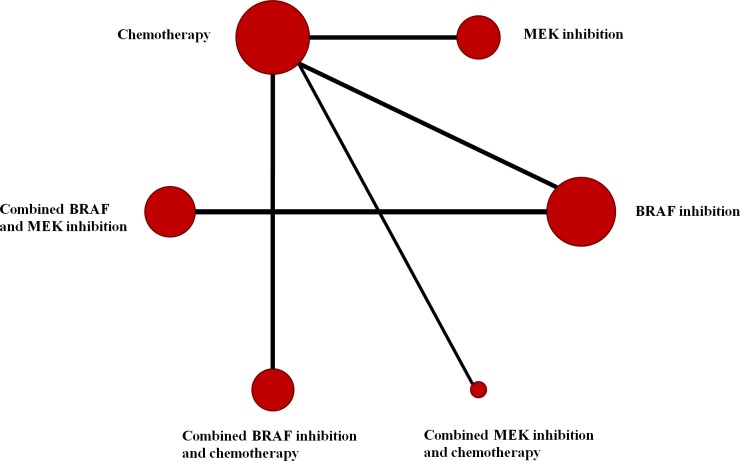
Network of comparisons for the Bayesian network meta-analysis Each circle represents an agent included in the analysis, with the area proportional to the number of studies comparing the particular arms. Each line represents direct comparisons between agents, with the thickness corresponding to the number of available direct within-trial comparisons.

We identified 451 relevant references for review title and abstract. After initial screening, we retrieved the full text of 32 potentially eligible clinical trials for detailed assessment. Of these, 22 randomized controlled trials were evaluated in more detail, and 18 randomized controlled trials with phase II or III were found that met the eligibility criteria for this study. Finally, 16 eligible trials reporting randomized controlled trials were included for meta-analysis, with a total of 5976 patients randomized to receive two of the six treatment strategies [1, 2, 5, 8-11, 13-21]. Figure 1 depicted the flow diagram of the systematic literature search and selection of random control trials. The characteristics of the 16 included trials were summarized in the Table 1. Six strategies were included: combined BRAF and MEK inhibition, combined BRAF inhibition and chemotherapy, combined MEK inhibition and chemotherapy, BRAF inhibition alone, MEK inhibition alone and chemotherapy alone. Figure 2 showed all the comparisons analyzed within the network. Across the 16 trials, BRAF mutant patients accounted for 64.45% (3851/5976).

**Table 1:** Characteristics of the eligible trials

	Year	Comparison		No. of Patients	Progress-free survival	Over-all survival	Response rate (%)		BRAF mutation	ClinicalTrials.gov number	Phase
		Arm 1	Arm2		HR (95%CI)	HR (95%CI)	Arm 1	Arm2			
**Carvajal^27^**	2014	Selumetinib	Temozolomide	101	0•46(0•30-0•71)	0•66(0•41-1•06)			Unknown	NCT01143402	II
**Chapman^1^**	2011	Vemurafenib	Dacarbazine	675	0•26(0•20-0•33)	0•37(0•26-0•55)	48	5	V600E	NCT01006980	III
**Flaherty^9^**	2012	Dabrafenib trametinib	Dabrafenib	108	0•39(0•25-0•62)	NA	76	54	V600E V600K V600R	NCT01072175	II
**Flaherty^5^**	2012	Trametinib	Dacarbazine Paclitaxel	322	0•42(0•29-0•59)	0•54(0•32-0•92)	22	8	V600E, V600K	NCT01245062	III
**Flaherty^22^**	2013	Sorafenib Carboplatin Paclitaxel	Carboplatin Paclitaxel	823	0•90(0•78-1•03)	1•01(0•87-1•18)	20•50	18•20	Unknown	NCT00110019	III
**Gupta^24^**	2014	Selumetinib Docetaxel	Docetaxel Placebo	83	0•75(0•50-1•14)	1•15(0•71-1•84)	32	14	None	NCT01256359	II
**Hauschild^21^**	2009	Sorafenib Carboplatin Paclitaxel	Carboplatin Paclitaxel placebo	270	0•91(0•63-1•31)	1•01(0•76-1•36)	12	11	Unknown	NCT00111007	III
**Hauschild^2^**	2012	Dabrafenib	Dacarbazine	250	0•30(0•18-0•51)	0•61(0•25-1•48)	50	6	V600E	NCT01227889	III
**Kirkwood^26^**	2012	Selumetinib	Temozolomide	200	1•07(0•86-1•32)	NA	5•80	9•40	BRAF NRAS	NCT00338130	III
**Larkin^10^**	2014	Vemurafenib cobimetinib	Vemurafenib Placebo	495	0•51(0•39-0•68)	0•65(0•42-1•00)	68	45	V600	NCT01689519	III
**Long^8^**	2014	Dabrafenib Trametinib	Dabrafenib	423	0•75(0•57-0•99)	0•64(0•42-0•95)	67	51	V600E, V600K	NCT01584648	III
**McArthur^25^**	2014	Vemurafenib	Dacarbazine	675	0•38(0•32-0•46)	0•70(0•57-0•87)	57	9	V600E, V600K	NCT01006980	III
**McDermot^20^**	2008	Sorafenib Dacarbazine	Dacarbazine placebo	101	0•67(0•43-1•03)	1•02(0•65-1•62)	24	12	Unknown	NCT00110994	II
**Ribas^28^**	2013	Trametinib	Chemotherapy	655	NA	0•88(0•77-1•05)	11	10	Unknown	NCT00257205	III
**Robert^23^**	2013	Selumetinib Dacarbazine	Dacarbazine placebo	91	0•63(0•47-0•84)	0•93(0•67-1•28)	40	26	Unknown	NCT00936221	II
**Robert^11^**	2015	Dabrafenib Trametinib	Vemurafenib	704	0•56(0•46-0•69)	0•69(0•53-0•89)	64	51	V600	NCT01597908	III

### Progress-free survival (PFS)

**Figure 3:**
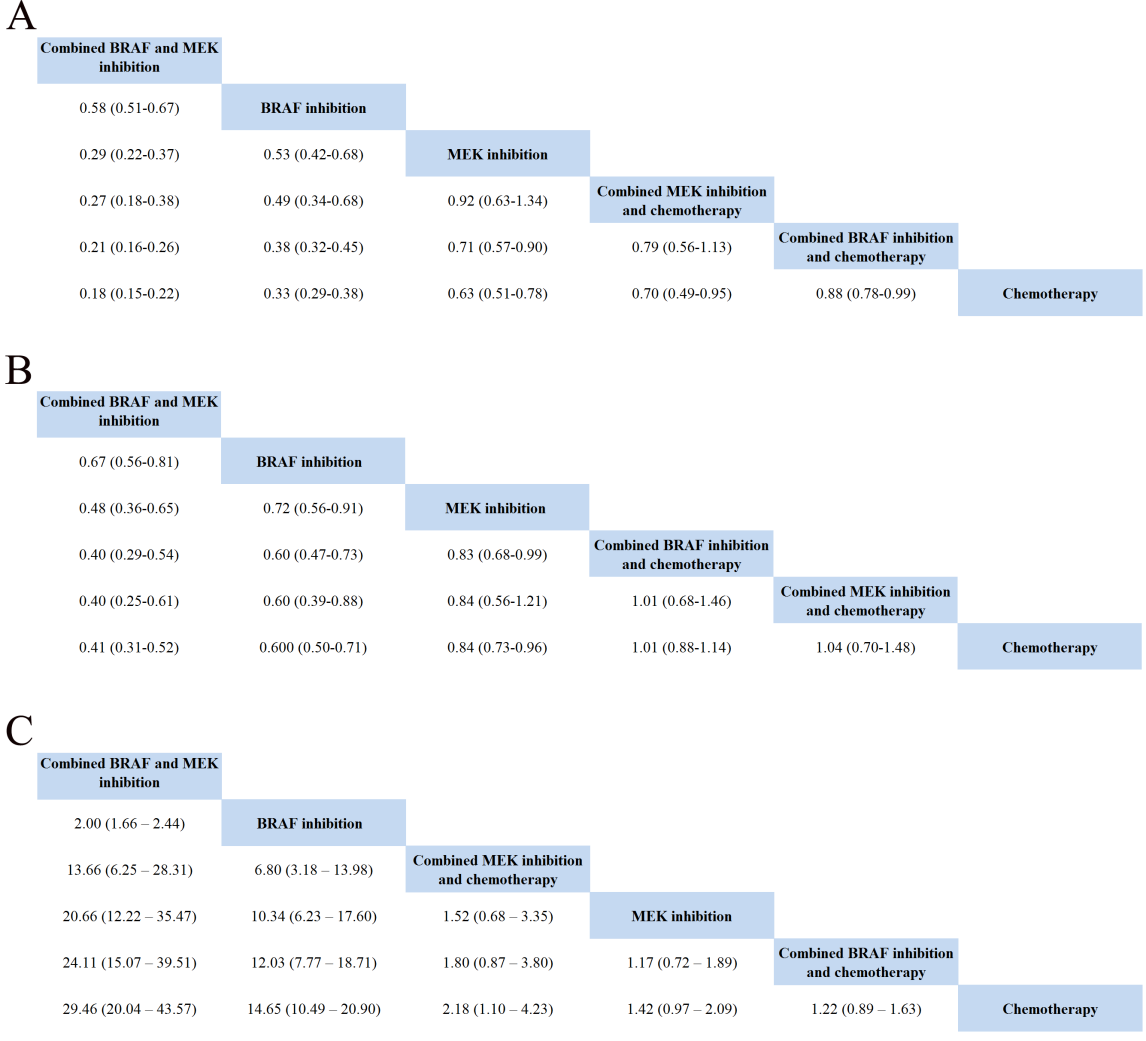
Pooled hazard ratios for survival and odds ratios for objective response rate by network meta-analysis

**Figure 4:**
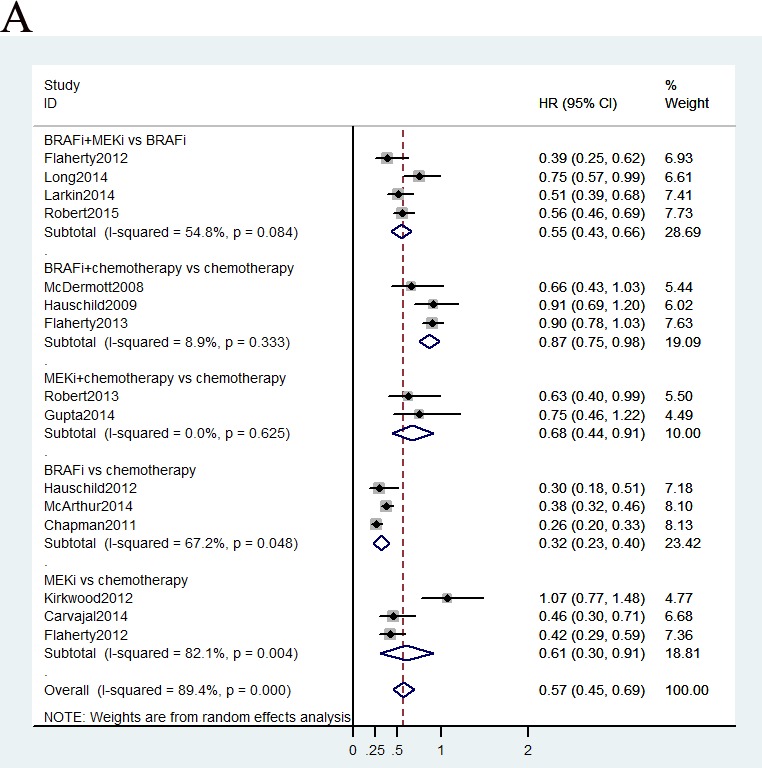

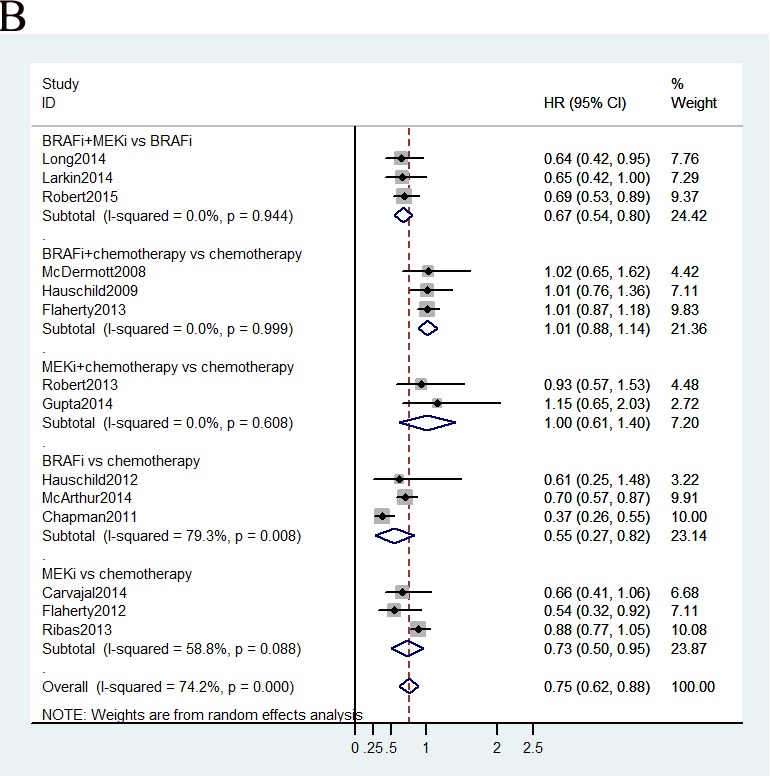

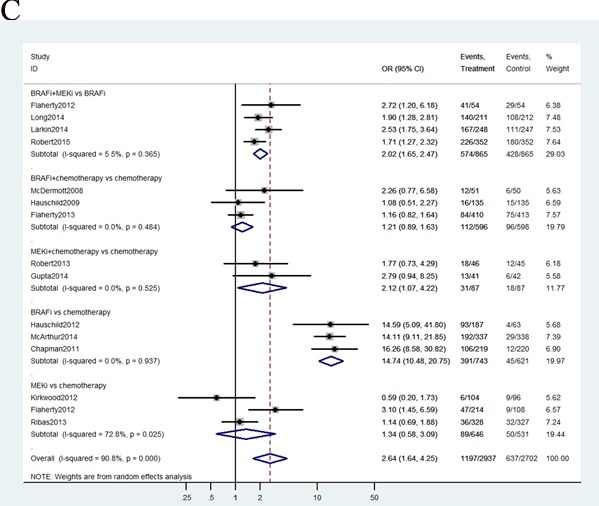
Pooled hazard ratios for survival and odds ratios for objective response rate by traditional meta-analysis

Data on PFS were available in fifteen studies, and HR values were explicitly reported in those studies. We summarized the results of our random-effects network meta-analysis for PFS in Figure 3A. Combined BRAF-MEK inhibition improved significant prolonged PFS, as compared with BRAF inhibition (HR: 0.58, 95%CI: 0.51-0.67, *P* < 0.0001) or MEK inhibition alone (HR: 0.29, 95%CI: 0.22-0.37, *P* < 0.0001), respectively. The network graph and forest plot of traditional pair-wise direct comparison were drawn to graphically display the results of the available direct comparisons between treatments. Comparing results from traditional pairwise meta-analysis (Figure 4A) and network meta-analysis did not suggest inconsistency between direct and indirect evidences. The network meta-analysis showed a statistically significant advantage for BRAF inhibition as compared with MEK inhibition (HR: 0.53, 95CI%: 0.42-0.68, *P* < 0.0001).

### Overall survival (OS)

Total 15 trials, with enrolled patients, contributed to our analysis of OS. As shown in Figure 3B, the ranking probabilities of treatment from the network meta-analysis of OS indicated that, of the 6 therapeutic strategies, combined BRAF-MEK inhibition had the highest probability of being the best treatment arm for MM. Combined BRAF-MEK inhibition improved significant prolonged OS comparing with BRAF inhibition (HR: 0.67, 95%CI: 0.56-0.81, *P* < 0.0001) or MEK inhibition alone (HR: 0.48, 95%CI: 0.36-0.65, *P* < 0.0001), respectively. Whereas, these results demonstrated that single BRAF inhibition had a statistically significantly longer in OS than MEK inhibition alone (HR: 0.72, 95%CI: 0.56-0.91, *P* = 0.008), and combined BRAF inhibition and chemotherapy (HR: 0.60, 95%CI: 0.47-0.73, *P* < 0.0001). This finding strengthened the results of the direct comparisons (Figure 4B).

### Objective response rate (ORR)

**Figure 5:**
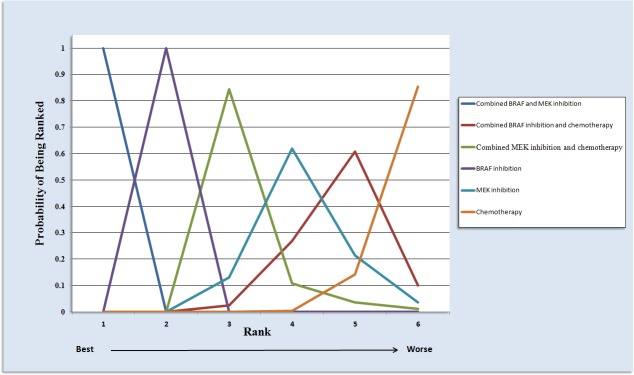
Ranking of treatments in terms of objective response rate by values of surface under the cumulative ranking probability curve

Total 15 studies including patients contributed to the analysis of objective response rate (ORR). In the Figure 3C, the strategy was better when corresponding OR value was over 1. Compared with chemotherapy, combined BRAF-MEK inhibition improved highest ORR (OR: 29.46, 95%CI: 20.04-43.57, *P* < 0.0001), followed by BRAF inhibition alone (OR: 14.65, 95%CI: 10.49-20.90, *P* < 0.0001), and combined MEK-chemotherapy (OR: 2.18, 95%CI: 1.10-4.23, *P* = 0.5982). Furthermore, the ORR was superior in patients who received combined BRAF-MEK inhibition compared with those who received BRAF inhibition (OR: 2.00, 95%CI: 1.66-2.44, *P* < 0.0001) or MEK inhibition alone (OR: 20.66, 95%CI: 12.22-35.47, *P* < 0.0001). The single BRAF inhibition yielded better response rate than MEK inhibition alone (OR: 10.34, 95%CI: 6.23-17.60, *P* < 0.0001). Values of surface under the cumulative ranking probability curve (SUCRA, Figure 5) indicated that combined BRAF and MEK inhibition had the highest probability of being the best treatment arm for ORR (SUCRA = 1.00), followed by BRAF inhibition alone (SUCRA = 0.80), and combined MEK and chemotherapy (SUCRA = 0.56). Analysis of inconsistency between direct (Figure 4C) and indirect comparisons indicated that no statistically significant inconsistency was identified in ORR.

## DISCUSSION

Despite major advancements in targeted therapy for MM, however, most patients relapse and show progressive disease after 7 months with treatment of BRAF or MEK inhibition alone. The crucial issue is how to combine targeted inhibition to maximize survival for patients with MM [22] and to ascertain whether early use of a combination of BRAF and MEK inhibitors is the best strategy to forestall resistance [23]. To address this issue, this meta-analysis provides for the first time a comprehensive assessment of the effectiveness of combined BRAF and MEK inhibition with PFS, ORR, and OS. Currently, the network meta-analysis supports the combined BRAF and MEK inhibition is the preferred strategy in patients with MM.

Different measures of survival can be combined in a single analysis on the HR scale, avoiding potential selection bias and loss of information due to only including studies with the same measure or doing separate analyses for different measures [24]. Network meta-analysis is a well established research method capable of comparing different trials using a common reference trial while maintaining the randomisation design [25]. Our network meta-analysis integrated evidence of HR and variation from direct and indirect comparisons while fully preserving randomisation within each trial. From this presented results, direct comparisons to each of the comparator categories were largely similar to the multiple comparison analyses. Specially, the results of network meta-analysis for survival indicated PFS were significantly prolonged in patients who received combined BRAF-MEK inhibition compared with those who received BRAF inhibition (HR: 0.58, 95%CI: 0.51-0.67, *P* < 0.0001) or MEK inhibition alone (HR: 0.29, 95%CI: 0.22-0.37, *P* < 0.0001), respectively. Combined BRAF-MEK inhibition also improved the OS over BRAF inhibition (HR: 0.67, 95%CI: 0.56-0.81, *P* < 0.0001) or MEK inhibition alone (HR: 0.48, 95%CI: 0.36-0.65, *P* < 0.0001). The similar benefit has been found in the ORR, which was superior in combined BRAF and MEK inhibition compared with BRAF inhibition (OR: 2.00, 95%CI: 1.66-2.44, *P* < 0.0001) or MEK (OR: 20.66, 95%CI: 12.22-35.47, *P* < 0.0001) inhibition alone. These data provide clear evidence for the benefit of BRAF-MEK inhibition combination therapy over BRAF or MEK inhibition alone in prolonging survival and higher response rate. This promising result of combined BRAF and MEK inhibition will allow physicians to select this concurrent inhibition as the preferred therapeutic strategy for patients with MM. We also believe this meta-analysis is the largest and most comprehensive study of initial targeted therapy for MM so far, and provides the highest level of evidence for patients with MM.

The survival benefit of combined BRAF and MEK inhibition, not only patients with MM but also those with operable BRAF mutation-positive melanoma has been reported previously [26]. Furthermore, combining immunotherapy (anti-CTLA4, anti-PD-1, and anti-PD-L1) and targeted therapy (BRAF and MEK inhibitors) may result in improved antitumor activity with the high response rates of targeted therapy and the durability of responses with immunotherapy. Addition of the MEK inhibitor trametinib would enhance the antitumor activity of combined immunotherapy with the BRAF inhibitor dabrafenib [27]. Therefore, our meta-analysis indicated the combined BRAF and MEK inhibition will be essential for maximizing clinical benefit of combining immunotherapy and targeted therapy. Investigation of the anti-tumor immune response such as CD8 T-cell-rich infiltrate during combined BRAF and MEK-targeted therapy can also yield novel therapeutic strategies [26]. Although the treatment modality is encouraged, the combined BRAF and MEK-targeted therapy is insufficient for long-term durable responses for MM. Increased MAPK reactivation in early resistance to dabrafenib/trametinib combination therapy of BRAF-mutant MM has been identified commonly via BRAF amplification and mutations activating NRAS and MEK2 [28]. Therefore, to maximize efficacy and overcome acquired resistance are challenges for rational conduct of clinical trials.

Prospective trials directly comparing single BRAF inhibition to single MEK inhibition are lacking. This meta-analysis is the first to assess the PFS, OS, and OSS between single BRAF and MEK inhibition, and fills a crucial knowledge gap of MAPK pathway. Our present results demonstrated that the single BRAF inhibition had a statistically significantly longer in PFS (HR: 0.53, 95CI%: 0.42-0.68, *P* < 0.0001), OS (HR: 0.72, 95%CI: 0.56-0.91, *P* < 0.0001), and higher OSS (OR: 10.34, 95%CI: 6.23-17.60, *P* < 0.0001) than those in MEK inhibition alone.

This study provides insight into the concurrent inhibition of BRAF and MEK for MM; however, it does have some limitations. First, 5 trials with irrespective of the BRAF mutation were included in this present meta-analysis. However, BRAF mutation status has been hypothesized to predict disease recurrence and response to chemotherapy in melanoma patients [29]. Identification and stratification of constitutively activating BRAF mutations in MM has led to observe homogeneous efficacy for different therapeutic strategies. Second, combined dabrafenib and trametinib was the first combined BRAF and MEK inhibition tested in clinical trials [9]. In our analysis, the combined BRAF and MEK inhibition was mixed by dabrafenib-trametinib and vemurafenib-cobimetinib combination. In the future, the characteristics of each combination, and comparison between different combination should be evaluated to identify best combinative inhibition [30]. Furthermore, studies evaluating the combination of BRAF/MEK inhibition with other inhibition such as PI3K/mTOR should be considered. Finally, an important consideration is that this study only analyzes efficacy for combined BRAF and MEK inhibition, in future study toxic effects should be evaluated when comparing those targeted therapies, such as the incidence of pyrexia [31], panniculitis [32], gastrointestinal or ocular toxicity cutaneous adverse events [33].

## CONCLUSIONS

Knowing all therapeutic options before therapy initiation will allow physicians to better plan targeted therapy options including sequence or combine inhibition. Given the impressive tumor ORR, PFS and OS, it is clear that combined BRAF and MEK inhibition improves upon and offers the maximum opportunity for those benefits in patients with MM. The first priority of therapeutic efficacy of combined BRAF and MEK inhibition also provides the robust cornerstone for future triple combination therapy of BRAF and MEK inhibitors with immunotherapy in patients with MM.

## MATERIALS AND METHODS

### Search strategy and selection criteria

We searched database of PubMed, the Cochrane Collaboration Central Register of Controlled Clinical Trials, Cochrane Systematic Reviews, and ClinicalTrials.gov for randomized controlled study without year and language restrictions, using the following search algorithm: combined targeted therapy AND melanoma. After the combinations of dabrafenib–trametinib or vemurafenib–cobimetinib have been identified, the keywords of individual inhibition of BRAF (dabrafenib, vemurafenib, sorafenib) and MEK (trametinib, cobimetinib, selumetinib), trial and melanoma were used to search relevant studies according to our previous MAPK therapy review [34].

First, the titles and abstracts of study reports have been identified by the search strategies for eligibility, and then full-text versions of all eligible studies were obtained for data synthesis. All randomized controlled trials that compared at least two arms of different treatment regimens involving targeted therapy were obtained. We required trials to include data for hazard ratio (HR) for PFS and OS, and conformed to the convention of reporting HR showing benefit of experimental drug versus control (HR < 1 favouring the experimental group and > 1 favouring the control group). The ORR defined as complete (CR) or partial response (PR) was according to Response Evaluation Criteria in Solid Tumors (RECIST).

### Data synthesis

Three investigators (Mai RQ, Zhou SX and Zhong WX) independently reviewed the full article of eligible trials and extracted information into an electronic database. From each eligible trial, the first author, year of publication, sample size, BRAF mutation, Clinical Trials.gov number, randomized phase and treatments of experimental and control group were recorded. Primary and secondary endpoints were also recorded. The primary end point was PFS. Secondary end points included OS and ORR, which were measured according to the Response Evaluation Criteria in Solid Tumors (RECIST) [35]. The reported HR was our preferred end point because HR account for censoring, provide time-to-event information [36]. When HR were not reported we estimated them from summary statistics with the method described by Tierney et al. [37]. We extracted the data for HR and corresponding 95% credibility intervals (CI) for PFS and OS analysis.

### Statistical analysis

The traditional pair-wise meta-analysis has been performed by Stata 12 (StataCorp, College Station, TX, USA) for PFS, OS and ORR, respectively. For network meta-analysis, the model applied to analyze the HR of PFS and OS was a Bayesian consistency model as described in Woods et al. [24], with 240000 iterations to obtain the posterior distributions of model parameters and 40000 burn-ins. The LnHR and SE were generated according to the HR and corresponding CI value described by Tierney et al. [37]. HR below one indicated a benefit of the experimental intervention. We compared ORR with odds ratio (OR) with 95% CI using NetMetaXL, which provides an interface for conducting a Bayesian network meta-analysis using WinBUGS 1.4.3 (MRC Biostatistics Unit, Cambridge, UK) from within Microsoft Excel [38]. To assess whether there was inconsistency between direct and indirect comparisons, the pooled HR from the network meta-analysis have been compared with corresponding HR from traditional pair-wise random-effects meta-analysis of direct comparisons as previous described by Liao [36]. Each analysis was based on non-informative uniform with random-effect model accounting heterogeneity among studies. We estimated 95% CI from the 2.5th and 97.5th percentiles of the posterior distribution. The *P* value from the 95% confidence interval has been evaluated according to the method described by Altman DG [39]. We did sensitivity analyses by repeating the main computations using a fixed-effect method. The reporting of this meta-analysis is based on the Preferred Reporting Items for Systematic Reviews and Meta-Analyses (PRISMA) guidelines [40].
